# Terahertz Spectroscopic Signatures of Microcystin Aptamer Solution Probed with a Microfluidic Chip

**DOI:** 10.3390/s19030534

**Published:** 2019-01-28

**Authors:** Mingkun Zhang, Zhongbo Yang, Mingjie Tang, Deqiang Wang, Huabin Wang, Shihan Yan, Dongshan Wei, Hong-Liang Cui

**Affiliations:** 1 School of Electrical Engineering and Intelligentization, Dongguan University of Technology, Dongguan, Guangdong 523808, China; zhangmk@cigit.ac.cn; 2 Chongqing Engineering Research Center of High-Resolution and 3D Dynamic Imaging Technology, Chongqing Institute of Green and Intelligent Technology, Chinese Academy of Sciences, Chongqing 400714, China; yangzhongbo@cigit.ac.cn (Z.Y.); mjtang@cigit.ac.cn (M.T.); dqwang@cigit.ac.cn (D.W.); wanghuabin@cigit.ac.cn (H.W.); yanshihan@cigit.ac.cn (S.Y.); 3 College of Instrumentation Science and Electrical Engineering, Jilin University, Changchun 130061, China

**Keywords:** terahertz, absorption signature, microfluidic chip, microcystin, molecular dynamics

## Abstract

Terahertz signature detection of biological samples in aqueous solution remains a great challenge due to the strong terahertz absorption of water. Here we propose a new preparation process for fabricating a microfluidic chip and use it as an effective sensor to probe the terahertz absorption signatures of microcystin aptamer (a linear single-stranded DNA with 60 nucleotides) dissolved in TE buffer with different concentrations. The microfluidic chip made of silicon includes thousands of 2.4 μm × 2.4 μm square-cross-section channels. One repeatable terahertz absorption signature is detected and recognized around 830 GHz, fitted to a Lorentz oscillator. This signature is theorized to originate from the bending of hydrogen bonds formed between adjacent hydrated DNA bases surrounded by water molecules. Furthermore, the low-lying vibrational modes are also investigated by molecular dynamics simulations which suggest that strong resonant oscillations are highly probable in the 815–830 GHz frequency band.

## 1. Introduction

Biomolecules can exhibit low-lying vibrational modes at the Terahertz (THz) frequency ranging from tens of gigahertz to several THz. Exploring characteristic THz absorption of biomolecules is particularly interesting because the detected signatures can be used as the spectroscopic fingerprints to differentiate biomolecules, which makes THz spectroscopy an effective and label-free analytical tool like IR and Raman spectroscopies, but with specificities particularly associated with conformational change and large-scale deformation of biomolecules, and interactions that are too low in frequencies to be detectable by IR and Raman spectroscopy. To probe the weak interactions such as hydrogen bonds and van der Waals interactions, THz wave has been employed in recent years to detect protein conformational change [1,2], biomolecular interaction [3,4], and DNA hybridization [5,6]. However, analyses of biological samples by THz spectroscopy have mainly focused on dehydrated or lyophilized samples to date. Given that proteins require water to function in living bodies, studying biomolecules in physiological solution has more practical significance. However, the use of THz spectroscopy to analyze aqueous samples has long been hampered by the strong THz absorption of water since the permanent dipole of water dominates and often masks any specific THz absorption which may occur from known biomolecules in solution [7,8].

To circumvent the “water problem”, several groups have recently begun adopting microfluidic chips as aqueous sample carriers to modulate THz wave transmission. Baragwanath [9] and George [10] et al. developed fully sealed microfluidic cells using high THz transmission materials. Not only the amount of the liquid sample to be measured is precisely controlled, but also the stability of THz spectral measurements is guaranteed. Lei et al. [11] proposed a quartz-based microfluidic cell with one main 50-μm deep and 1-cm wide channel and multiple inlets/outlets to investigate the multi-stream mixing dynamics of sequentially injected fluids via THz mapping. Shimul et al. [12,13] fabricated a microfluidic device based on a silicon grating structure with the grating pattern period of 20–35 μm. Due to the smaller THz loss of silicon relative to the polar liquid, the overall THz wave transmission in the microfluidic device was higher, and thus the signal strength and available bandwidth were improved significantly. Since microfluidic techniques can precisely control the quantity of the liquid sample to be measured, ensure the equivalent THz optical path among multiple measurements, and effectively reduce water absorption of THz wave, the marriage of THz wave and microfluidic devices has been widely pursued to analyze hydration dynamics of chemical and biological liquids [9,10,11,12,14,15,16], distinguish analogues of biomolecules [13,17] and probe the hydration shell thicknesses of proteins [18,19].

In addition to exploring the hydration of biomolecules via THz absorption intensity, some theoretical studies as well as our previous simulations have confirmed that biomolecules in solution can produce characteristic resonant absorption in THz band [20,21]. However, large absorption caused by water molecules has always been an obstacle to discovering resonant signatures in aqueous solution. Moreover, compared with intermolecular vibrations of crystal structures, the vibrational modes of dissolved biomolecules are much more diverse and stochastic, making it extremely difficult to observe the obvious, stable, and reproducible THz signatures in aqueous solution. In order to reduce the background absorption of water and restrain some vibrational modes of biomolecules in solution, Brown et al. [22,23] dispersed biomolecular solutions in a sub-micrometer (800–1200 nm) channel array and adopted a coherent photomixing spectrometer with a high frequency resolution of less than 1 GHz to measure the THz absorption signatures of DNA and RNA molecules. Several absorption signatures for the small-interfering RNA (20–25 bp) in TPE buffer with a concentration of 9 ng/μL [23] and λ-DNA (48.5 kbp) in EDTA buffer with a concentration of 0.5 μg/μL [22] were observed from 0.8 to 1.1 THz. Similarly, we have fabricated a silicon-glass microfluidic chip to revisit the THz signatures of dissolved λ-DNA in TE buffer of 0.3 μg/μL and also found absorption signatures around 850 and 950 GHz [24]. These research efforts served to authenticate that the fine pitch channel array can effectively reduce the absolute absorption due to the reduction of water quantity for THz transmission detection, while accentuating vibrational resonances of biomolecules by the confinement of the channels [22,23,25,26]. 

In this paper, we report on our effort to detect THz absorption signatures of microcystin aptamer in TE buffer by using silicon glass sealed microchannels as an effective sensor. The microcystin aptamer is a synthetic single-stranded DNA (ssDNA) with 60 nucleotides. It has been widely used for highly affinitive and specific detection of microcystin in solution [27,28,29]. The attenuated signals in THz transmission spectra were fitted with the Lorentz oscillator model, and the resonant fingerprint was distinguished by the fitting parameters. Finally, the low-lying eigen-frequencies and oscillator strengths of microcystin aptamer in water were analyzed and explained via molecular dynamics simulations and quasi-harmonic approximation.

## 2. Materials and Methods

### 2.1. Microfluidic Chip

**Figure 1:**
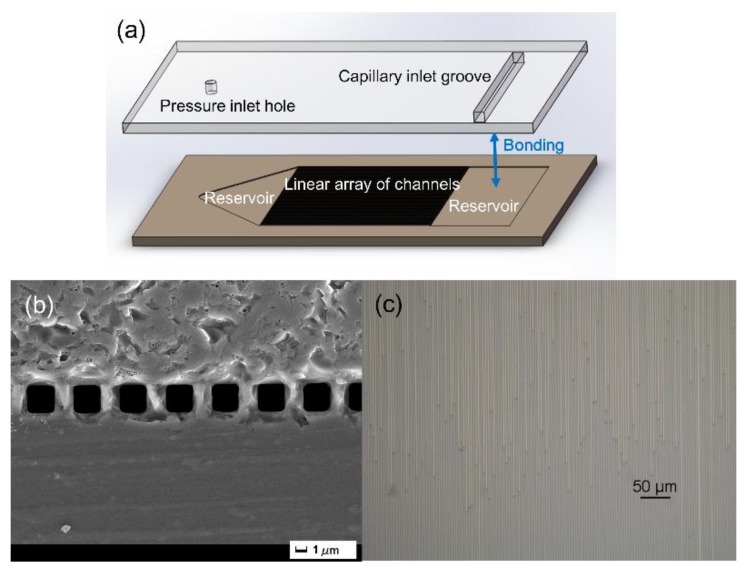
(**a**) An exploded view of the chip which is 38.5 mm long, 22.0 mm wide, and 1.0 mm thick. The channel array has an area of 16.0 × 16.0 mm^2^ which is comparable to the size of the incident THz light spot. There are around 4,000 flow channels lying parallel between two reservoirs. (**b**) SEM end view of the 2.4 μm wide channels etched on the silicon substrate and bonded with the glass film. (**c**) Optical micrograph of top view of microchannels being injected with liquid.

The microfluidic chip is composed of a 470-μm thick single-crystal silicon wafer with a resistivity above 3.5 kΩ·cm attached with a 500-μm thick silica glass sheet by anodic bonding, as depicted in Figure 1a. High-resistivity silicon is almost transparent to THz radiation, and it is also suitable for semiconductor processing. Silica glass also has a good THz transmission and is transparent in the visible light region, which allows us to conveniently observe the liquid flow pattern in microchannels. Each microchannel is 2.4 μm wide and 2.4 μm deep and the pitch between two channels is 1.6 μm. The SEM image of the sealed microchannel array is shown in Figure 1b. The fabrication procedure of this microfluidic chip includes the following steps: (1) Produce the reservoirs and the linear array of microchannels on a mask plate via laser direct writing lithography; (2) Spin coating of AZ1500 photoresist on a silicon wafer, and then transfer the mask pattern to the silicon wafer via interferometric lithography; (3) Etch the structure to a given depth by using reactive ion etching technique, and then wash the residual photoresist to fully clean the substrate; (4) Mark the inlet and outlet positions on the upper glass film and bore a hole or groove with an appropriate size in the marked location; (5) Combine the silicon wafer and the glass film by using the anodic bonding technology [30] to form an enclosed chip. More detailed description of the chip is available from our previous work [24].

A Bruker A140-H sample holder is used to carry the chip in the measurements. The sample holder consists of two parts: the pedestal and the clamp. The pedestal is fixed in the optical path and remained stationary in all measurements. The clamp and the pedestal are connected by magnet rivets. The microfluidic chip is embedded and tightened on the pedestal during the measurement and it is removed from and reloaded on the pedestal when changing the measured targets. The magnet rivet link on the sample holder can guarantee the microfluidic chip to be placed almost on the same position on the pedestal.

Before each measurement, the liquid sample is first injected into the capillary inlet groove shown in Figure 1a, and disperses over the inlet reservoir, then it flows into the channels due to the capillary force. The advancing flow fronts in channels constantly penetrate forward over time. An instantaneously captured micrograph of flow fronts is shown in Figure 1c. The liquid finally fills all channels and penetrates into the outlet reservoir. The penetration in each channel is nearly uniform and equivalent after a few minutes of the capillary action. 

### 2.2. Coherent Photomixing Spectrometer

**Figure 2:**
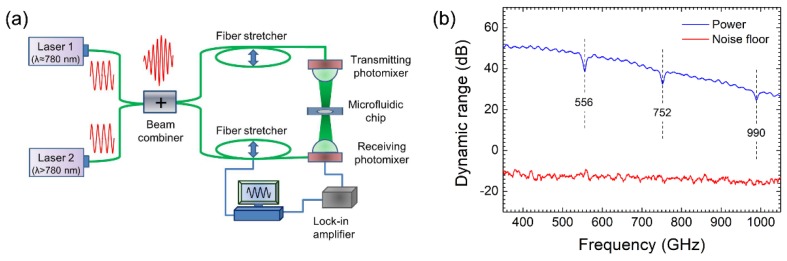
(**a**) Schematic of THz coherent photomixing spectrometer setup and the location of the microfluidic chip adjacent to the photomixers. (**b**) Variations of transmission power, background noise with frequency. Water vapor absorptive attenuations are marked with dashed vertical lines.

To measure the characteristic signatures of dissolved biomolecules more sensitively, a coherent photomixing spectrometer (TeraScan 780, TOPTICA Photonics AG, Munich, Germany) was adopted owing to its high spectral resolution and superior dynamic range. A block diagram of the spectrometer is shown in Figure 2a. Two temperature-controlled distributed feedback (DFB) lasers with a center wavelength of about 780 nm are directed into a 50:50 coupler and splitter for achieving two optical beams whose envelope frequency is located at the THz band. One beam is transmitted to the ion-implanted GaAs photomixer for generating THz radiation and the other is transmitted to the similar ion-implanted GaAs photomixer for coherently detecting the THz signal attenuation caused by the absorption of the sample in microchannels. Two fiber stretchers are added to the optical beam for directly detecting the amplitude and phase in a certain frequency range. The spectrum is obtained by sweeping the single-frequency THz transmit photomixer over a specified frequency range from 0.05 to 2.0 THz with an instantaneous line width in MHz magnitude [31]. 

To verify the superior dynamic range of the THz coherent photomixing spectrometer, the power response from 0.4 to 1.0 THz and the noise floor obtained when the THz beam is blocked but all other settings are kept normal are shown in Figure 2b. The ratio of the power response to the noise floor is 61 dB at 0.4 THz, 54 dB at 0.7 THz, and 43 dB at 1.0 THz, respectively. In addition, the narrow absorption features of water vapor are clearly observed at 556, 752, 990 GHz, which are in agreement with the report in Ref. [31].

### 2.3. Sample Preparation and Spectrometer Adjustment

The microcystin aptamer is a linear ssDNA with the sequence of 5’-GGCGCCAAAC AGGACCACCA TGACAATTAC CCATACCACC TCATTATGCC CCATCTCCGC-3’ [27,28,29] and molecular weight of 18167 g/mol. The aptamer was purchased from Sangon Biotech Company (Shanghai, China). The DNA aptamer was dispersed in TE buffer (10 mM Tris-HCl and 1mM EDTA) with two concentrations of 0.92 and 0.23 μg/μL, respectively. A holder was first placed in the light path between two photomixers as shown in Figure 2a, then the holder position was fine-tuned to ensure the THz radiation through the aperture is as large as possible. In measurements, the coherent beam was tuned continuously from 0.4 to 1.0 THz with a discrete frequency step of 350 MHz. The ambient temperature is 23 °C and the humidity is controlled lower than 50%.

The prepared samples with the two different concentrations of 0.92 and 0.23 μg/μL were measured in turn. For each measurement, the dry chip was first put on the holder to measure the THz transmission power of the blank chip and this spectrum was recorded as the blank signal, $P_{B}\left(v\right)$. The beam spot of the spatially coherent THz beam is usually a circular dot with a radius of several millimeters, while the size of the channel array in the chip is 16×16 mm^2^, so THz wave only radiates on the sealed microfluidic channels. Secondly, ~0.6 μL TE buffer was injected into the chip to fully immerse the channels in 1–2 minutes by the capillary force and the referenced signal $P_{R}\left(v\right)$ was recorded. Thirdly, deionized water and nitrogen were injected into the chip in turn with a fluidic pressure pump to fully rinse and blow dry the channels. Fourthly, ~0.6 μL DNA solution was injected into the chip smoothly in 5–8 minutes. After the full injection, the THz signal of DNA solution in the microfluidic channels $P_{S}\left(v\right)$ was recorded. Lastly, the chip was rinsed with TE buffer and deionized water in turn and dried with the nitrogen flow again for the next measurement. 

### 2.4. Molecular Dynamics Simulation

To obtain the THz absorption signatures of the microcystin aptamer in theory, molecular dynamics simulations of microcystin aptamers in aqueous solution were performed with AMBER12 software [32]. The oscillator strengths and the absorption spectrum of the microcystin aptamer from 400 to 1000 GHz were calculated and analyzed by the quasi-harmonic approximation [20,21]. Firstly, the 60-bp ssDNA was established and Na^+^ ions were added to neutralize the negative charges of the ssDNA within the ff12SB force field [33]. Secondly, the energy minimization in vacuum was performed and then the system was heated to 298 K with Langevin dynamics for 10 ns to obtain a stable configuration of ssDNA in vacuum. Thirdly, the ssDNA was solvated in an explicit TIP3P water box of 6.2 × 7.8 × 21.7 nm^3^ with periodic boundary conditions. 1936 atoms of the ssDNA, 59 Na^+^ ions, and 34082 water molecules were included in the simulation system. The solution system was then energy minimized and conducted in the NPT ensemble with a standard atmospheric pressure of 1.0 bar at 298 K for 2 ns. After the NPT simulation, a stable density reached 1.001 g/cm^3^. Lastly, the system ran under NVT ensemble for 10 ns for equilibrium and the subsequent product analysis. The time step of all simulations is 2.0 fs.

Ten different configurations without dynamic correlation were selected as the initial configurations from the above NVT simulation after equilibrium for the product simulations. The duration of each product simulation was 90 ps [20,21] which matches the time scale of hydration relaxation dynamics of most biomolecules [34]. The individual trajectory was recorded every 0.2 ps, resulting in a 450-frame trajectory file for the calculation of the oscillator strengths of the ssDNA in solution.

## 3. Results

### 3.1. THz Signature Estimation from Transmission Spectra

**Figure 3:**
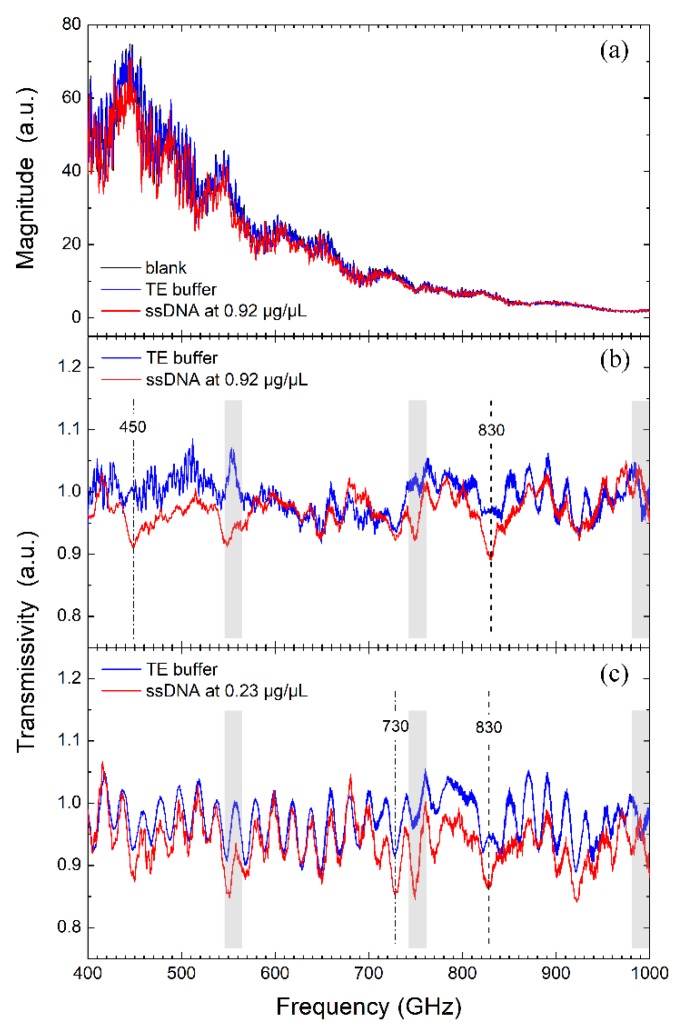
(**a**) Transmission spectra of the blank chip, the TE buffer and the ssDNA at 0.92 μg/μL; Transmission spectra of the microfluidic chip containing pure TE buffer relative to the blank chip (blue curve), and the microfluidic chip containing DNA solutions relative to the blank chip (red curve) at 0.92 μg/μL (**b**) and at 0.23 μg/μL (**c**).

THz transmission spectra of the blank chip, the TE buffer and the ssDNA at 0.92 μg/μL are shown in Figure 3a. From Figure 3a, we can see that the transmission intensity has a small change when the measured target changing from the blank chip to the TE buffer solution since the depth of the microchannel is very thin (2.4 μm). We also see that there is a small difference between the transmission intensities of the TE buffer and the ssDNA solution. To further analyze the difference of these two transmission spectra, the transmissivities of the TE buffer relative to the blank chip $T_{R}\left(v\right)=P_{R}\left(v\right)/P_{B}\left(v\right)$ and the DNA solution relative to the blank chip $T_{S}\left(v\right)=P_{S}\left(v\right)/P_{B}\left(v\right)$ are plotted against frequency in blue and red lines, respectively as displayed in Figure 3b,c at different concentrations. In this experiment, since the thickness of the grooved channels is only 2.4 μm, the interactive path length between THz wave and the liquid in microchannels is one order of magnitude less than the thickness of the liquid film in most liquid measurements [9,12]. When the chip is full of liquid, it does not cause a significant full-band amplitude attenuation, and hence all the THz transmissivities at different frequencies are close to 1. In addition, the transmission of DNA solution is generally lower than that of the TE buffer, which is consistent with the previous THz spectroscopy measurements of the 20-nt ssDNA [8] and 13-nt ssDNA [35]. 

It can be seen that near 550 GHz, there is an obvious convex peak for the TE buffer curve and a concave valley for the ssDNA solution in Figure 3b, which are the noises caused by absorption signature of ambient water vapor at 556 GHz as described in Section 2.2. Since the minor change of the ambient humidity in the experimental environment will lead to the obvious change of the absorption amplitudes of water vapor, so when calculating the transmissivities of *T*_R_ and *T*_S_, there are some big fluctuations near the water vapor signatures. Therefore, any undulation less than 20 GHz around the water vapor signatures of 556, 752, 990 GHz which are marked with gray shadows in Figure 3b,c is considered as a disturbance and excluded from the signature recognition. It is worth noting that the red line has two obvious attenuations compared to the blue line around 450 and 830 GHz in Figure 3b. For pure TE buffer and ssDNA solution, they differ only in whether containing the ssDNA component, so the attenuation may be attributed to the signatures of ssDNA in aqueous solution. 

Next, we changed the concentration of the ssDNA solution to check whether the attenuated signatures at the present concentration still appear at a lower concentration of 0.23 μg/μL. Measurements were performed under the same conditions as those at 0.92 μg/μL. Results show that the transmissivity of the ssDNA solution is still generally lower than that of the pure TE buffer as shown in Figure 3c. When DNA molecules dissolve in aqueous buffer, hydration shells are formed around the solute molecules, the coupling between DNA and hydration water alters the collective water network dynamics in the hydration layer. Since the hydration water usually represents stronger absorption than the bulk water [36,37], the transmissivities of solutions decrease when DNA is dissolved. 

It is worth noting that the transmission curve of the TE buffer shown in Figure 3c is not exactly consistent with that in Figure 3b. There are more undulations in the transmission curve at 0.23 μg/μL compared to that at 0.92 μg/μL. The difference may come from the internal reflections in the spectrometer as well as the slight spatial shifts in the sample location along the path of the beam since the load and unloading of the microfluidic chip are controlled by hand. To some extent, the identification of the characteristic absorption features is affected because the undulations are of the same order of magnitude as the intensity of characteristic absorptions. We still observed the THz absorption signature at 830 GHz. In addition, there appear other possible features such as the one at 730 GHz, but they are ambiguous because the buffer curve also has similar attenuation signatures at those frequencies.

### 3.2. Signature Recognition via Fitting Analysis

**Figure 4:**
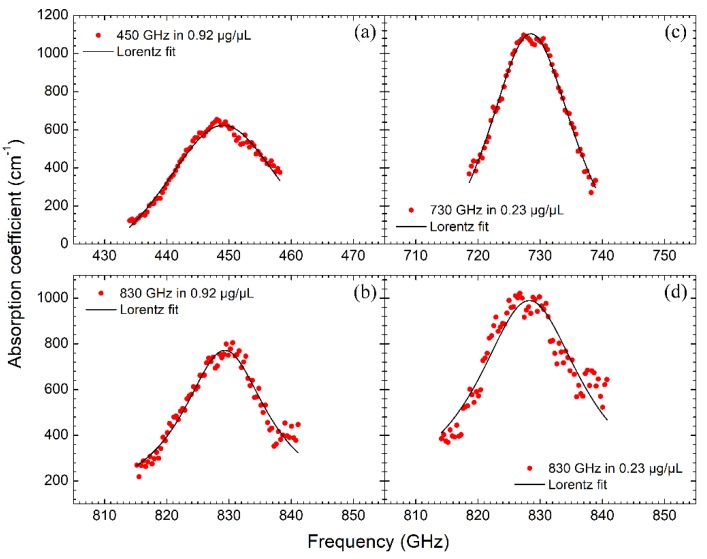
The fitting of absorption coefficients around the attenuated signals with the Lorentz function, (**a**,**b**) represent the 450 and 830 GHz attenuations at 0.92 μg/μL, (**c**,**d**) represent the 730 and 830 GHz attenuations at 0.23 μg/μL.

In this section, we recognized the possible resonant signatures amongst the attenuated signals mentioned in the above section via a data fitting analysis. The absorption coefficients α(υ) in the vicinities of these signatures can be calculate from Lambert–Beer law,

(1) $$\alpha \left(\upsilon \right)=\frac{1}{fd}\ln\;T_{S}\left(v\right)$$

where f=2.4/(2.4+1.6)=0.6 is the liquid fill factor which can be estimated from the channel and grating pitch widths, *d* is the thickness of the measured liquid of 2.4 μm. The absorption signals were fitted with the Lorentz oscillator line profile [38,39] as,

(2) $$\alpha \left(\upsilon \right)=\alpha_{b}+S_{k}\frac{\upsilon^{2}\gamma }{{\left(\upsilon^{2}-\upsilon_{k}^{2}\right)}^{2}+\upsilon^{2}\gamma^{2}}$$

where α(υ) is the background attenuation, *S*_k_ is the oscillator strength in the *k* th mode, $\upsilon_{k}$ is the resonant frequency, γ is the oscillator dissipative factor which numerically equals to the full width at half maximum (FWHM) of the fitted peak. Variations of the absorption coefficient with frequency for several possible THz signatures and the corresponding numerical fitting curves are depicted in Figure 4. The fitting parameters from each curve are listed in Table 1.

From Table 1, we can see that the background attenuations of the resonances centered at 449.1 GHz at 0.92 μg/μL and 728.5 GHz at 0.23 μg/μL are negative, which violates the optical absorption law and should be removed from the resonant signatures. While the fitting data of the resonant peaks at 829.3 GHz at 0.92 μg/μL and 828.3 GHz at 0.23 μg/μL look reasonable. The background attenuations and the oscillator strengths from these two peaks are comparable to 165 cm^−1^ and 518.1 cm^−2^ of the 13-nt ssDNA, respectively [35], and the FWHMs also coincide with 10–50 GHz of the 15–25 bp double-stranded RNAs [23,26,35]. These results further support the hypothesis that the characteristic signature near 830 GHz is associated with the ssDNA molecules. In addition, it is worth noting that the THz absorption of the ssDNA solution decreases with the increase of the ssDNA concentration, especially at the resonant frequency, as shown in Figure 4b,d and seen from the fitted oscillator strength in Table 1.

### 3.3. Resonance Analysis Using Quasi-Harmonic Approximation

The low-lying vibrational modes of the ssDNA in solution are investigated by molecular dynamics simulations, and the quasi-harmonic approximation is used to estimate the oscillator strength of the ssDNA [20,21]. In the quasi-harmonic approximation, the harmonic potential model is constructed from a position fluctuation matrix [40], given by

(3) $$\sigma =\langle \left(q-\langle q\rangle \right){\left(q-\langle q\rangle \right)}^{T}\rangle =k_{B}T\cdot F^{-1}$$

where ***q*** is the mass-weighted atomic position vector, 〈⬚〉 express the atomic vibrational equilibrium positions, *F* is the force constants matrix, *k*_B_ is the Boltzmann constant, *T* is the thermodynamic temperature. The elements in σ can be calculated directly from the atomic coordinates of molecular dynamics.

The variable dipole moment in the *k* th mode of an *N*-atom molecule is calculated as

(4) $$p^{k}=\overset{N}{\underset{i=1}{\sum }}\frac{e_{i}a_{ki}}{\sqrt{m_{i}}}$$

where the subscript *i* denotes atomic index, *e*_i_ is the atomic partial charge, *m*_i_ is the atomic mass, and $a_{ki}$ is a displacement vector extracted from the eigenvectors. Then the oscillator strength *S*_k_ is calculated as

(5) $$S_{k}=\frac{{\left(p^{k}\right)}^{2}}{\sum_{i}^{N}m_{i}a_{ki}^{2}}$$

hence, the absorption spectrum in the full bandwidth can be obtained by introducing each oscillator strength of the *k* th mode into Equation (2) and summing over all the modes.

**Figure 5:**
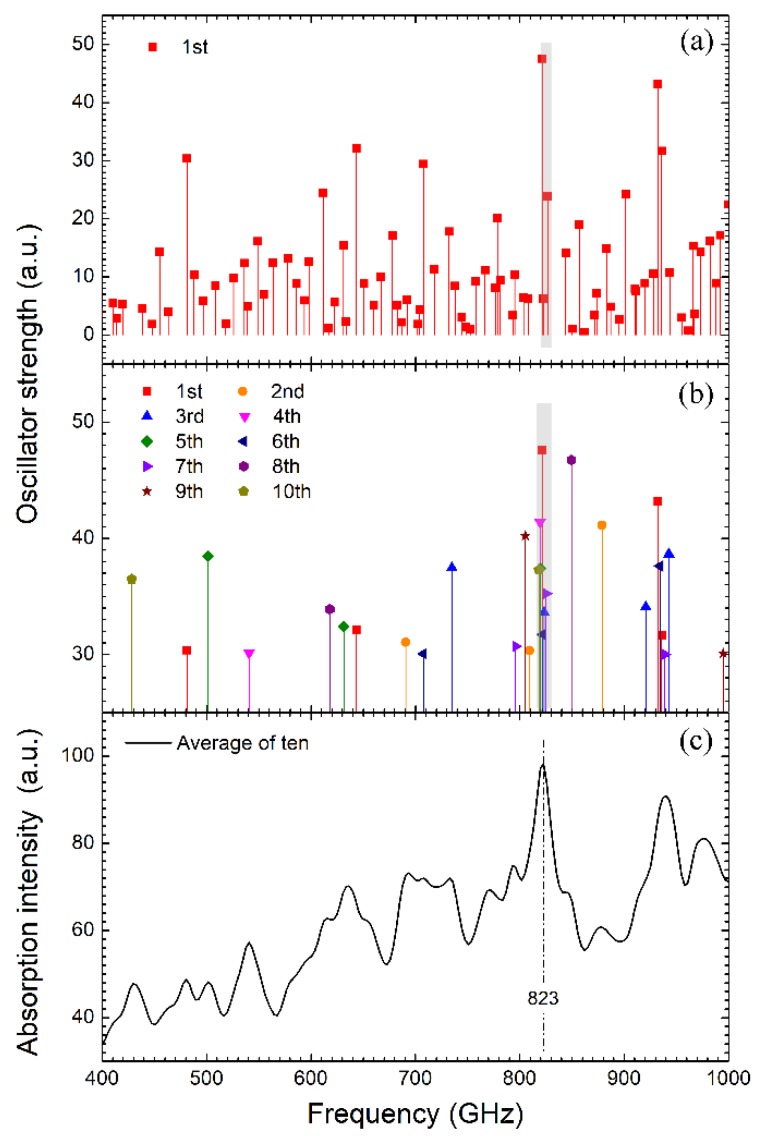
(**a**) Oscillator strengths of the 1st product simulations of the ssDNA in water calculated by the quasi-harmonic approximation; (**b**) Oscillator strengths larger than 30 a.u. in the total ten product simulations; (**c**) Averaged absorption spectrum of the ten product simulations.

The oscillator strengths calculated from the first of the ten product simulations of the ssDNA in solution are shown in Figure 5a. Three oscillator strengths fall in the 820–830 GHz frequency band, which are highlighted with a gray shadow. Other oscillator strengths populate almost the entire frequency band. Theoretically, 3*N*-6 (*N* is the atom number of the ssDNA) vibrational modes with 3*N*-6 eigenvalues and eigenvectors can be obtained from THz to infrared bands, but not all of these vibrational modes can be detected in experiment. Therefore, Figure 5b omits the small oscillator strengths and only shows the ones larger than 30 a.u. for all the ten simulations. From Figure 5b, all of the ten simulations have oscillator strengths larger than 30 a.u. in the frequency region of 800–850 GHz, and seven of them occur in the very narrow 815–830 GHz band, marked also with a gray shadow, which indicates that the probability of strong resonance appearing around 830 GHz is very high. In Figure 5c, all the oscillator strengths of the ten simulations were superimposed via the Lorentz function (Equation (2)) with a dissipative factor γ of 0.6 cm^-1^ which is the averaged value of the fitting parameter in the above experiments. When the weak resonances are filtered, the strongest resonant absorption estimated theoretically stands at 823 GHz in Figure 5b, which does not deviate much from the 830 GHz signature measured in the experiment. 

For the ssDNA molecule, connections between the phosphate groups, sugar rings, and bases are via covalent bonds with the force constants about 0.1–0.6 mdyne/Å, which lead to the eigen-frequencies of covalent modes falling almost entirely in IR and even higher bands. While the adjacent bases are connected via hydrogen bonds with the force constant about 0.025 mdyne/Å. Therefore, the THz vibrational modes around 830 GHz originate most likely from the hydrogen bonds bending between the adjacent bases. For dry DNA, the lowest vibrational mode appears around the 40 cm^−1^ (1.2 THz) [41]. On the other hand, for dissolved DNA, the effect of hydration on the harmonic dynamics gives rise to the mass loading of the DNA molecular subunits by binding water molecules, which causes the vibration to shift to a lower frequency [35].

## 4. Conclusions

In this work, we designed and fabricated a microfluidic chip with thousands of channels via a series of lithography and bonding processes. The microfluidic channels can avoid liquid evaporation during the measurements, be washed using a pressure pump for repeated use. We probed the THz absorption signatures of the microcystin aptamer with 60 nucleotides dissolved in TE buffer at 0.92 and 0.23 μg/μL concentrations by using this chip and a coherent photomixing THz spectrometer. Experimental results showed that one remarkable signature around 830 GHz repeatedly appeared, which was subsequently identified by a Lorentz oscillator fitting. Furthermore, the strongest resonant mode of the dissolved microcystin aptamer at 823 GHz was predicted by molecular dynamics simulations and the quasi-harmonic approximation, which corroborated the experimental finding nicely. 

This work presents a study of THz absorption signatures of DNA molecules in aqueous solution. Our study indicates that THz spectrometer combined with a microfluidic chip can be used as an effective label-free technique to detect THz spectroscopic signatures of biomolecules in solution. 

## Figures and Tables

**Table 1:** The fitting parameters of each Lorentz curve in Figure 4.

	Resonant Center (GHz)	Oscillator Strength (cm^−2^)	Background Attenuation (cm^−1^)	FWHM (GHz)
Figure 4a	449.1 ± 0.1	890.1 ± 109.0	−345.9 ± 64.7	27.6 ± 1.7
Figure 4b	829.3 ± 0.1	377.3 ± 46.1	94.3 ± 38.7	16.7 ± 1.3
Figure 4c	728.5 ± 0.1	890.4 ± 93.7	−346.2 ± 80.8	18.4 ± 1.0
Figure 4d	828.3 ± 0.2	600.1 ± 144.6	111.0 ± 106.1	20.5 ± 2.8
